# The Dosage of Chloroform

**Published:** 1900-02-03

**Authors:** 


### THE DOSAGE OF CHLOROFORM.

Writing on the subject of deaths from chloroform Dr. James Edmunds[1] maintains that in the administration of that drug we ought to look for the same precision of dosage as that which we ensure when we inject morphia or strychnine. Dispensers there are, he says, who could measure out a milligramme of strychnine on the end of a knife more accurately than others could weigh it, and the same is the case with 20 milligrammes of morphia. But we insist on our hypodermic syringes being graduated, and of our solutions being of a strength defined by the balance and the burette, and we do not allow pharmaceutical geniuses to play "heads or tails" with the lives of our patients by guessing out strychnia on the end of a knife. That chloroform in vapour is a powerful poison is demonstrated by the number of deaths which occur every year from its use. Nevertheless, chloroform is, perhaps, the most beneficent of all drugs, and in order to be made safe it only needs to be administered with precision as to its dosage, and not by rule-of-thumb methods such as geniuses believe that they can safely practise. By the use of a proper apparatus it is now possible to administer chloroform with the same precision as we have long secured for the administration of other drugs. "Geniuses who have acquired the habit of slopping chloroform about by the ounce, and who only kill a patient now and then will, of course, be disgusted at having to acquire methods of order and precision such as will ensure a comfortable and safe anæsthesia with one-tenth of the chloroform now used. The volumetric dosage of anæsthetic vapours will, in fact, be received in the same way as the stethescope and the clinical thermometer were sometimes received. But the volumetric dosage of anæsthetic vapours will come to stay." The inventive genius of instrument makers has been lavish in the elaboration of the details, but so far as principles are concerned it would appear that up to the present time it is only by the use of one or other of the several modifications of the Junker apparatus that measured and precise administration of chloroform can be secured. Dr. Edmunds ties himself down to the advocacy of one particular apparatus, but without going so far as that, which indeed is not necessary, it remains the fact that the only practical modes of administering measured quantities of chloroform vapour at present before the profession are derivatives of the Junker.

[1] Lancet, Jan. 27.

